# Garlic-derived allyl sulfides attenuate house dust mite-induced allergic airway inflammation associated with reduced PI3K/NF-κB signaling

**DOI:** 10.3389/fphar.2026.1906382

**Published:** 2026-09-04

**Authors:** Hsu-Chung Liu, Keng-Fan Liu, Mann-Jen Hour, Chia-Chen Hsieh, Wei-Sung Li, Hong-Zin Lee, Jen-Chieh Tsai

**Affiliations:** 1 Department of Medicine Division of Chest Medicine, Chung-Kang Branch, Cheng Ching Hospital, Taichung, Taiwan; 2 Division of Plastic Surgery, Department of Surgery, Kaohsiung Medical University Chung-Ho Memorial Hospital, Kaohsiung, Taiwan; 3 School of Pharmacy, China Medical University, Taichung, Taiwan; 4 Department of Pharmacy, China Medical University Beigang Hospital, Yunlin, Taiwan; 5 Cancer Biology and Precision Therapeutics Center, China Medical University, Taichung, Taiwan; 6 Department of Medicinal Botanicals and Foods on Health Applications, Da-Yeh University, Changhua, Taiwan; 7 Plant Pathology Division, Taiwan Agricultural Research Institute, Ministry of Agriculture, Taichung, Taiwan

**Keywords:** asthma, Der p, house dust mite, immune modulation, PI3K/NF-κB, S-allyl-l-cysteine

## Abstract

**Background:**

Allergic asthma, characterized by chronic airway inflammation, is often triggered by *Dermatophagoides pteronyssinus* (Der p). Garlic-derived allyl sulfides, including diallyl trisulfide (DATS), diallyl disulfide (DADS), and *S*-allyl-L-cysteine (SAC), possess anti-inflammatory properties, yet their effects on allergic asthma remain unclear. This study aimed to investigate the protective effects of these compounds on Der p-induced allergic asthma and to elucidate their underlying mechanisms *in vivo* and *in vitro*.

**Methods:**

BALB/c mice were sensitized and challenged to develop allergic asthma and administered with DATS, DADS, or SAC. Histological phenomena were examined using hematoxylin and eosin (H&E) staining. Bronchoalveolar lavage fluid (BALF) was analyzed for pro-inflammatory (TNF-α, IL-1β, IL-12) and anti-inflammatory (IL-10, TGF-β) cytokines, as well as Th1/Th2 markers (IFN-γ, IL-4, IL-5, and IL-13). Serum immunoglobulins, including IgE, IgG1, and IgG2a subclasses, were quantified. Parallel experiments using A549 alveolar epithelial cells examined PI3K/NF-κB signaling via Western blot analysis.

**Results:**

Among the tested compounds, SAC showed the strongest effects, significantly reducing inflammatory cell infiltration, restoring Th1/Th2 balance by elevating IFN-γ and reducing IL-4, IL-5, and IL-13, and lowering TNF-α, IL-1β, and TGF-β while enhancing IL-10. SAC also decreased total IgE and IgG1 while increasing IgG2a, suggesting a shift toward Th1-mediated immunity. In A549 cells, SAC inhibited Der p-induced PI3K and IKK expression and suppressed Akt and NF-κB phosphorylation, thereby reducing IL-6 expression. Both *in vivo* and *in vitro* findings, complemented by structural molecular visualization, highlight SAC as the most effective for preventing and managing allergic asthma.

**Conclusion:**

These findings demonstrate that SAC alleviates Der p-induced airway inflammation by modulating immune responses and downregulating PI3K/Akt/NF-κB signaling, highlighting its potential as a natural therapeutic agent for allergic asthma.

## Graphical Abstract

**Figure:**
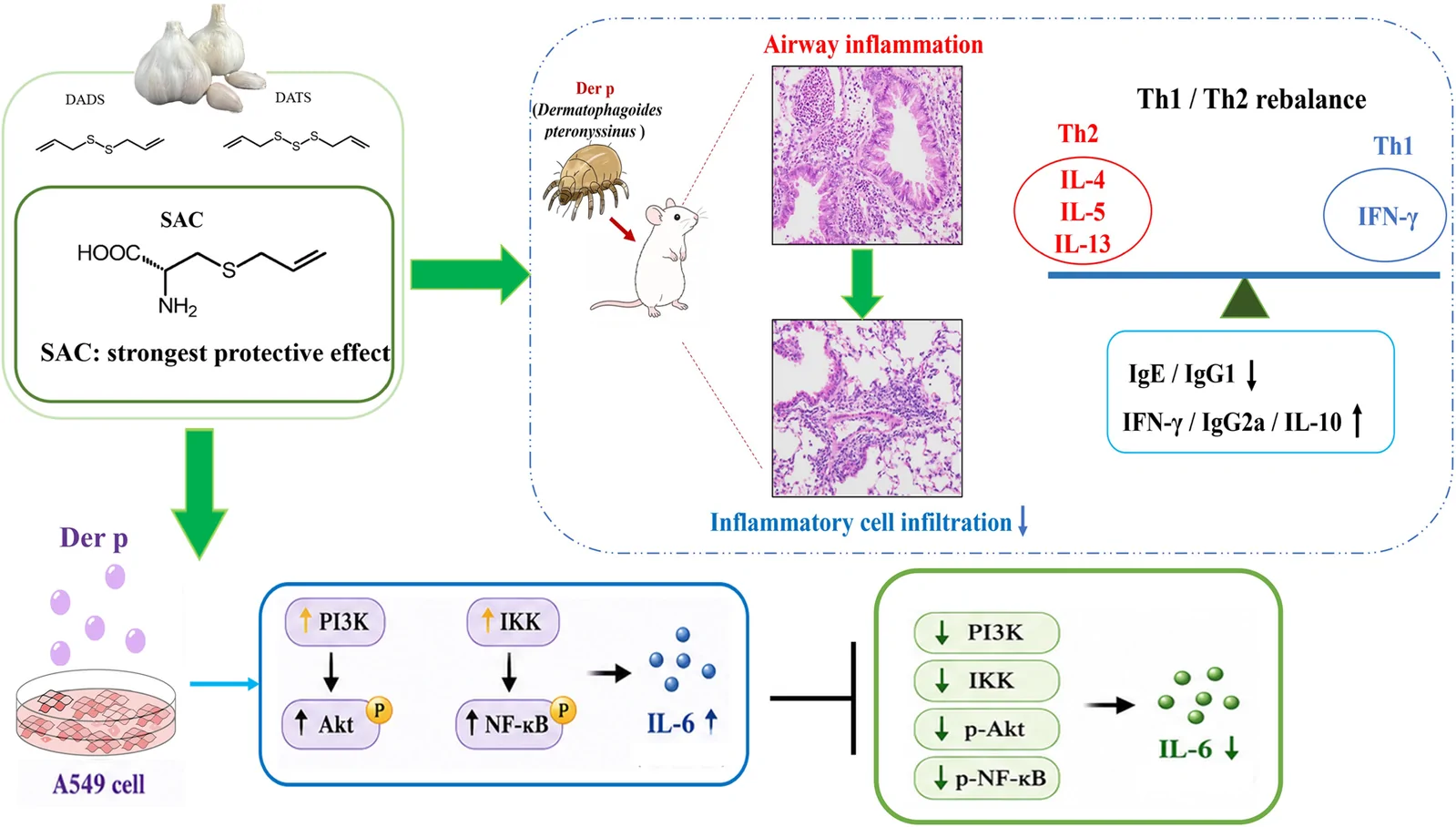

## 1 Introduction

Garlic (*Allium sativum* L.) contains a variety of bioactive components, most notably organosulfur compounds, which are responsible for its characteristic odor and many of its health benefits. These compounds include alliin, allicin, diallyl sulfide (DAS), diallyl disulfide (DADS), diallyl trisulfide (DATS), and *S*-allyl cysteine (SAC). Allicin, a highly reactive and unstable intermediate, is enzymatically generated from alliin by alliinase when garlic is crushed or chopped and subsequently decomposes into oil-soluble sulfides, including DAS, DADS, and DATS. On the other hand, SAC is a major water-soluble derivative found in garlic extracts (Colín-González et al., 2012). These compounds exhibit diverse pharmacological properties. SAC reportedly has antioxidant, anti-inflammatory, anticancer, antidiabetic, and hepatoprotective effects (Rais et al., 2023). Moreover, SAC reduced eosinophilic airway inflammation, T helper 2 (Th2) cytokine production, immunoglobulin E (IgE) levels, and mucus overproduction in an ovalbumin-induced allergic asthma model (Shin et al., 2019). Similarly, DADS reduced airway inflammation through activation of the nuclear factor erythroid 2-related factor 2 (Nrf2)/heme oxygenase-1 (HO-1) pathway and suppression of nuclear factor kappa B (NF-κB) signaling in the same model (Shin et al., 2013). DADS also exhibits antioxidant, anti-inflammatory, antimicrobial, anticancer, cardioprotective, and neuroprotective activities (Song et al., 2021). DATS has been reported to alleviate pulmonary inflammation and reduce tumor necrosis factor-α (TNF-α) and interleukin-6 (IL-6) production in H9N2 influenza virus-infected A549 cells and mice (Ming et al., 2021). Although previous studies have demonstrated the anti-inflammatory and immunomodulatory effects of SAC and DADS in ovalbumin-induced allergic asthma, their comparative effects have not been systematically evaluated in a house dust mite-induced allergic asthma model.

Allergic asthma is an inflammatory disease characterized by airway inflammation, reversible airflow obstruction, airway hyperresponsiveness, and airway remodeling (Akar-Ghibril et al., 2020). Due to airway hypersensitivity and inflammation, the airways constrict and produce excessive mucus, leading to symptoms such as wheezing, coughing, chest tightness, and difficulty breathing. In allergic asthma, exposure to allergens triggers an immune-mediated inflammatory response in the airways, particularly by Th2 cells, which release cytokines that cause airway inflammation, mucus production, and bronchoconstriction (Hammad and Lambrecht, 2021). House dust mites, particularly *Dermatophagoides pteronyssinus* (Der p), are among the most prevalent indoor allergens worldwide and are major contributors to allergic asthma. Der p allergens, particularly Der p 1 and Der p 2, can disrupt epithelial tight junctions, activate protease-activated receptors (PARs), and trigger innate immune signaling (Wan et al., 1999; Saito et al., 2021).

When stimulated by inflammatory signals, macrophages release cytokines that modulate immune responses, including pro-inflammatory mediators such as TNF-α, IL-1β, IL-6, and monocyte chemoattractant protein-1 (MCP-1), as well as anti-inflammatory cytokines such as IL-10 and transforming growth factor-β (TGF-β). TNF-α, IL-1β, IL-6, IL-8, and MCP-1 are among the major cytokines released by airway macrophages in asthma and play central roles in sustaining airway inflammation and recruiting immune cells (Herbert et al., 2010; Lee et al., 2015; Britt et al., 2023). Macrophages and dendritic cells phagocytose allergens and activate T cells, leading to differentiation of Th1 and Th2 cells. Th1 cells primarily produce IL-2, TNF-β, and interferon-γ (IFN-γ), whereas Th2 cells secrete IL-4, IL-5, and IL-13. Allergic asthma is characterized by a Th2-dominant cytokine imbalance that promotes airway inflammation and hyperresponsiveness (Barnes, 2001; Truyen et al., 2006; Durrant and Metzger, 2010). IL-4 is essential for inducing IgG1 and IgE class switching in B cells, and elevated IL-4 levels are closely associated with increased IgG1 and IgE production in allergic diseases (Snapper et al., 1988; Gour and Wills-Karp, 2015; Cernescu et al., 2021). IgG2a production depends on the Th1 cytokine IFN-γ and is typically associated with Th1-skewed immune responses (Bossie and Vitetta, 1991; Schüler et al., 1999).

Multiple signaling pathways, including phosphoinositide 3-kinase (PI3K)/Protein kinase B (Akt), MAPK, JAK/STAT, NF-κB, and Nrf2, have been implicated in the pathogenesis of allergic asthma. Among these, the PI3K/Akt signaling pathway has emerged as a key regulator of airway inflammation, immune regulation, and airway remodeling (Wang et al., 2023; Song et al., 2025). Activation of PI3K leads to downstream Akt signaling and subsequent activation of nuclear factor-κB (NF-κB), thereby promoting the transcription of pro-inflammatory cytokines and contributing to airway inflammation and remodeling (Rao et al., 2017; Zhang et al., 2017; Wang et al., 2022; Song et al., 2025). Moreover, PI3K signaling facilitates the recruitment and activation of immune cells, including eosinophils, neutrophils, macrophages, and dendritic cells, thereby sustaining airway inflammation (Lee et al., 2006; Datler et al., 2019). NF-κB activation is closely associated with Th2-type inflammation and IgE production in airway epithelial and immune cells (Baldwin, 1996; Poynter et al., 2004; Gong et al., 2012). Inhibition of PI3K/Akt and NF-κB signaling has been shown to reduce airway inflammation and IgE levels in murine asthma models (Ma et al., 2021). Given the central role of PI3K/Akt/NF-κB signaling in allergic airway inflammation, we investigated whether garlic-derived allyl sulfides could modulate this pathway.

Our previous study demonstrated that garlic water extract effectively attenuates Der p-induced allergic asthma in a mouse model by modulating Th1/Th2-related cytokine expression. HPLC analysis further indicated that *S*-allyl cysteine (SAC) may be the principal active component in the extract (Hsieh et al., 2019). Although SAC and DADS have shown protective effects in ovalbumin-induced allergic asthma models, the comparative anti-inflammatory effects and underlying mechanisms of SAC, DADS, and DATS in house dust mite-induced allergic asthma remain unclear. Therefore, this study aimed to compare the anti-inflammatory and immunomodulatory effects of SAC, DADS, and DATS in Der p-induced allergic asthma using *in vitro* and *in vivo* models and to investigate their potential molecular mechanisms through molecular docking analysis.

## 2 Materials and methods

### 2.1 Reagents

Chemical reagents, including DADS, DATS, and SAC, were obtained from Sigma-Aldrich (St. Louis, MO, United States). A commercially available *D. pteronyssinus* (Der p) extract was purchased from Allergon AB (Ängelholm, Sweden). Antibodies against Akt, p-Akt, NF-κB, p-NF-κB, PI3K, and IL-6 were sourced from Abcam (Cambridge, United Kingdom).

### 2.2 Experimental animals

Specific pathogen-free male BALB/c mice (6–8 weeks old) were obtained from BioLASCO Taiwan Co., Ltd. Animals were housed in an SPF facility under controlled conditions (23 °C ± 2 °C, 40%–60% humidity, 12-h light/dark cycle) with *ad libitum* access to standard chow and water. All procedures adhered to internationally recognized ethical guidelines for laboratory animal care and were approved by the Institutional Animal Care and Use Committee of Da-Yeh University (Permit No. 105033).

### 2.3 Experimental design and induction of Der p-induced asthma

After 1 week of acclimatization, BALB/c mice were weighed and randomly divided into five experimental groups (n = 8 per group). Group allocation was performed after confirming that there were no obvious differences in body weight among the animals. The sample size was selected based on our previous study using the same Der p-induced allergic asthma model (Hsieh et al., 2019). The control and Der p groups received water, while the remaining groups received SAC, DADS, or DATS (80 mg/kg) orally once daily for 4 weeks, starting 2 weeks before the first allergen sensitization and continuing until the allergen challenge. Allergic sensitization and challenge were performed as previously described (Hsieh et al., 2019). The dosage was chosen based on our previous research findings and literature (Hsieh et al., 2019; Khajevand-Khazaei et al., 2019). SAC, DADS, and DATS were administered at the same dose to compare their anti-inflammatory and immunomodulatory effects. Sensitization was performed on days 14 and 21. All mice except those in the control group received a subcutaneous injection at the base of the tail with 50 μL of emulsion containing 50 μg of Der p antigen in incomplete Freund’s adjuvant (Difco, Detroit, MI, United States). On day 28, mice were weighed and anesthetized with tiletamine/zolazepam (Zoletil® 50, Virbac Corporation, Carros, France; 20 mg/kg body weight, intraperitoneal injection) before the intranasal allergen challenge. For the challenge, 50 μL of Der p solution (1 mg/mL) was administered intranasally, and the mice were held upright for 1 min to facilitate inhalation. Control mice received 50 μL of saline. Seventy-two hours after the allergen challenge, mice were deeply anesthetized with tiletamine/zolazepam (Zoletil® 50, 20 mg/kg body weight, intraperitoneal injection). Blood was collected by cardiac puncture for serum preparation, and the mice were euthanized by exsanguination. BALF, lungs, and tracheal tissues were then collected.

### 2.4 Histological preparation of lung tissues

After euthanasia, the chest cavity was opened, and the lungs were inflated by introducing 70% ethanol-formalin solution through the trachea. The lungs were then excised, immersed in the same solution overnight, and subsequently transferred to 10% neutral buffered formalin for further fixation. Following fixation, the tissues were dehydrated with graded alcohol, embedded in paraffin, and sectioned into 4 μm slices using a microtome. Finally, the tissue sections were mounted on slides and stained with hematoxylin and eosin for analysis (Tanaka et al., 2001). Histopathological evaluation was performed by an investigator blinded to the treatment groups. The severity of lesions in the lungs was graded according to the methods described by Shackelford et al. (2002). The degree of lesions was graded from one to five depending on severity: 1 = minimal (<1%); 2: slight (1%–25%); 3 = moderate (26%–50%); 4 = moderately severe (51%–75%); 5 = severe/high (76%–100%). Mean histopathological scores were calculated by dividing the sum of the score per grade of affected mice by the total number of examined mice.

### 2.5 Preparation of BALF and inflammatory cell counting

Seventy-two hours after the last Der p challenge, mice were anesthetized and euthanized. Bronchoalveolar lavage was performed by gently washing the lungs three times with 1 mL of ice-cold sterile saline using a syringe. The collected lavage fluid was kept on ice and centrifuged at 12,000 rpm for 10 min at 4 °C. The resulting supernatant, designated as BALF, was stored at −80 °C for subsequent analysis, while the cell pellet was resuspended in PBS for total cell counting using a hemocytometer.

### 2.6 Measurement of cytokine concentrations in BALF

Cytokine concentrations in BALF were measured in duplicate using ELISA kits from BioSource (Camarillo, CA, United States) for Th1/Th2 cytokines and Abcam (Cambridge, United Kingdom) for macrophage cytokines, following the manufacturers' instructions. The absorbance was read on a Synergy™ HT Multi-Mode Microplate Reader (BioTek, Winooski, VT, United States).

### 2.7 Measurement of IgE, IgG1, and IgG2a concentrations in mouse serum

Following weighing, mice were anesthetized via an intraperitoneal injection of tiletamine/zolazepam (Zoletil® 50, Virbac Corporation, Carros, France; 20 mg/kg body weight). Anesthesia was confirmed by the loss of pain reflexes. Blood was then collected by cardiac puncture. The whole blood sample was transferred to a test tube without an anticoagulant and allowed to clot at room temperature for 1 hour. Serum was then isolated by centrifugation at 10,000 rpm for 10 min. Serum samples were stored at −70 °C until ready for the measurement of total IgE, IgG1, and IgG2a. IgE, IgG1, and IgG2a concentrations were measured using ELISA kits (Promega, Madison, WI, United States) according to the manufacturer’s instructions. The absorbance was read on a Synergy™ HT Multi-Mode Microplate Reader (BioTek, Winooski, VT, United States).

### 2.8 Preparation of lung tissue lysates for protein analysis

Lung tissues were homogenized in lysis buffer (50 mM Tris-HCl, pH 7.4; 1% NP-40) containing 1% protease inhibitor cocktail (Sigma-Aldrich, St. Louis, MO, United States). The homogenates were incubated on ice for 30 min and then centrifuged at 13,500 rpm for 20 min at 4 °C. The supernatant was collected, and the protein concentration was quantified using a BCA protein assay kit (Pierce Biotechnology, Rockford, IL, United States).

### 2.9 Cell culture

A549 cells obtained from the Food Industry Research and Development Institute (Hsinchu City, Taiwan) were cultured in DMEM/F12 medium supplemented with 10% fetal bovine serum (FBS), 1% L-glutamine, 1% HEPES, and 1% penicillin-streptomycin-amphotericin (PSA). Cells were maintained at 37 °C in a CO_2_ incubator and grown in 10-cm culture dishes. When cells reached 80%–90% confluence, they were subcultured to promote proliferation (Hsieh et al., 2019).

### 2.10 Cell viability assay

Cells were seeded at 1 × 10^6^ cells/well in 96-well plates and incubated at 37 °C in a 5% CO_2_ atmosphere for 24 h. Following incubation, cells were stimulated with Der p (5 μg/mL) and treated with SAC (0.1, 0.5, or 1 mM) for an additional 24 h under the same conditions. Cell viability was assessed by mitochondrial reductase activity using the MTT assay (3-[4,5-dimethylthiazol-2-yl]-2,5-diphenyltetrazolium bromide; Sigma-Aldrich, St. Louis, MO, United States). After treatment, the medium was replaced with serum-free medium containing 0.5 mg/mL MTT (pH 7.4), and cells were incubated for 2 h at 37 °C in the dark. The formazan crystals were dissolved in dimethyl sulfoxide (DMSO), and absorbance was measured at 570 nm using a microplate reader (BIO-TEK PowerWave × 340, Winooski, VT, United States). Cell viability (%) was calculated as the absorbance ratio of treated to control cells multiplied by 100.

### 2.11 Western blotting

Western blot analysis was conducted to evaluate the expression of PI3K and NF-κB in lung tissues and PI3K, Akt, p-Akt, IKK, NF-κB, p-NF-κB, and IL-6 in A549 cells. β-Actin was used as an internal control. For Western blot analysis, A549 cells were stimulated with Der p (5 μg/mL) and treated with DATS, DADS, or SAC (1 mM) for 24 h. Total proteins of A549 cells were extracted using a lysis buffer containing protease and phosphatase inhibitors (Sigma-Aldrich, St. Louis, MO, United States), and concentrations were determined with a BCA protein assay kit (Pierce Biotechnology, Rockford, IL, United States). Equal amounts of protein (50 μg) were separated on SDS-PAGE gels and transferred to PVDF membranes (Millipore, Billerica, MA, United States). The blot was blocked for 1 h in TBST (Tris-buffered saline: 10 mM Tris-HCl, 150 mM NaCl, 0.05% Tween-20) containing 3% bovine serum albumin (BSA), then incubated overnight at 4 °C primary antibodies against PI3K, Akt, p-Akt, IKK, NF-κB, p-NF-κB, and IL-6 in TBST. After washing three times (10 min each) with TBST, membranes were incubated for 2 h at room temperature with HRP-conjugated secondary antibodies in TBST, followed by another three washes in TBST. Protein bands were visualized using an enhanced chemiluminescence detection kit (PerkinElmer, Boston, MA, United States). The Western blot results of the protein were quantified by Lane 1D Gel imaging analysis software (Sagecreation, Beijing, China).

### 2.12 Molecular modeling

The three-dimensional crystal structure of the NF-κB p65 homodimer (PDB ID: 2RAM) was downloaded from the RCSB Protein Data Bank website (https://www.rcsb.org/structure/2RAM). The docking was performed with the CDOCKER program within the software package Discovery Studio 2021 (Accelrys, San Diego, CA, United States). S-allyl cysteine (SAC), diallyl disulfide (DADS), and diallyl trisulfide (DATS) were docked into 2RAM using the co-crystal ligand ((2S,3S)-1,4-dimercaptobutane-2,3-diol) as the binding site.

### 2.13 Biostatistical analysis

The data are presented as the mean ± standard error of the mean (SEM) and were analyzed using IBM SPSS Statistics (SPSS Inc., Chicago, IL, United States). Statistical differences among multiple groups were assessed using one-way analysis of variance (ANOVA), followed by Duncan’s multiple range test for *post hoc* pairwise comparisons. No formal statistical tests for normality or homogeneity of variance were performed. No data points were excluded as outliers unless they were attributable to identifiable technical or experimental errors. A p-value <0.05 was considered statistically significant.

## 3 Results

### 3.1 Effects of DATS, DADS, and SAC on BALF total inflammatory cells and airway histopathology in Der p-induced allergic asthma

**FIGURE 1:**
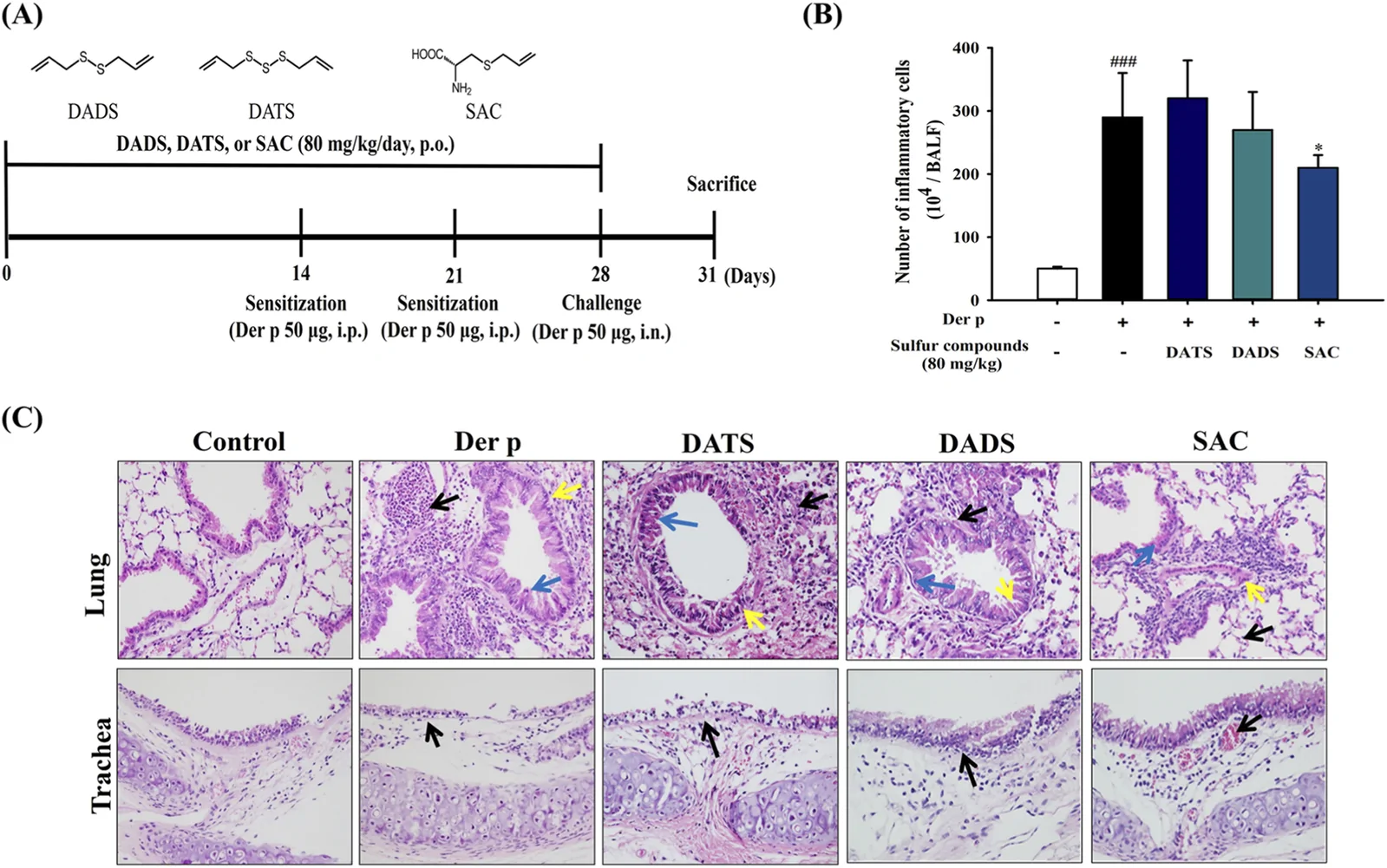
Effects of diallyl disulfide (DADS), diallyl trisulfide (DATS), and S-allyl cysteine (SAC) on Der p-induced airway inflammation. **(A)** Schematic of the allergic sensitization and challenge experimental protocol. i.p: intraperitoneal injection; i.n.: intranasal administration. **(B)** Total inflammatory cell counts in bronchoalveolar lavage fluid (BALF) from unsensitized controls, Der p-sensitized controls, and sensitized mice treated with DATS, DADS, or SAC (80 mg/kg). Data are expressed as mean ± SEM (n = 8 per group). ###P < 0.001 compared to unsensitized control; *P < 0.05 compared to sensitized control. **(C)** Representative hematoxylin–eosin (H&E) staining of lung (upper panel) and tracheal (lower panel) tissues (n = 4). Control mice exhibited normal alveolar and bronchial architecture without evident inflammatory infiltration. Der p exposure induced marked inflammatory cell infiltration and epithelial thickening. Treatment groups showed varying degrees of attenuation of inflammatory infiltration, with SAC demonstrating the most consistent reduction. Representative sections were examined at 400× magnification.

Der p is a prevalent allergen that triggers asthma and is widely used to induce Th2-dominant airway inflammation in experimental asthma models. The Der p-induced animal model closely resembles the typical characteristics of human asthma (Klein et al., 2020). In this study, DATS, DADS, and SAC were evaluated for their protective effects in a Der p-induced allergic asthma model (Figure 1A). To evaluate airway inflammation in Der p-induced allergic asthma, total inflammatory cells in BALF were quantified (Figure 1B). Dust mite sensitization markedly increased the total inflammatory cell count compared with control mice. SAC treatment significantly reduced the number of total inflammatory cells (P < 0.05), whereas DATS and DADS did not produce significant reductions.

Histopathological examination using hematoxylin–eosin (H&E) staining further supported these findings (Figure 1C; Table 1). In the control group, mice exhibited normal alveolar and bronchial architecture without evident inflammatory infiltration. Der p exposure resulted in marked inflammatory cell infiltration and epithelial thickening in both lung and bronchial tissues. As quantified in Table 1, the inflammation scores for peribronchial and perivascular infiltration were significantly elevated in the Der p group. Treatment with garlic-derived sulfide compounds was associated with varying degrees of reduction in inflammatory infiltration. DATS-treated mice showed that inflammatory cell infiltration remained apparent, with limited reduction compared to the Der p group. DADS treatment attenuated these pathological changes, with reduced epithelial thickening and inflammation compared with the vehicle group. SAC treatment produced the most pronounced improvement, demonstrating a significant restoration of bronchial epithelial structure and reduction in inflammatory infiltration scores (Table 1). A similar trend was observed in bronchial tissues, where SAC showed the most consistent attenuation (Figure 1C; Table 1). Specifically, SAC effectively attenuated the Der p-induced epithelial thickening, suggesting a direct protective effect on airway structural integrity. In summary, SAC showed the most significant improvement in airway inflammation and histopathological changes, while DADS had moderate protective effects, and DATS had limited effects.

**TABLE 1:** Quantitative histopathological scoring of lung and tracheal lesions in Der p–induced allergic asthma following treatment with diallyl disulfide (DADS), diallyl trisulfide (DATS), or S-allyl cysteine (SAC).

Organ	Histopathological findings^1^	Group				
		Control	Der p	Der p		
				DATS	DADS	SAC
Lung	Inflammation, lymphocytic and eosinophilic cells, perivascular and peribronchial, multifocal.	0.8 ± 0.50	4.0 ± 0.82^#^	3.5 ± 0.58	3.3 ± 0.96	1.5 ± 0.58^*^
	Aggregation, macrophage and giant cells, alveolar, focal	1.0 ± 0.00	4.5 ± 0.58^#^	3.8 ± 0.50	3.8 ± 0.50	2.0 ± 0.82^*^
	Epithelial hyperplasia, bronchial, focal	1.0 ± 0.82	4.0 ± 0.82^#^	3.8 ± 0.96	3.5 ± 1.29	2.3 ± 0.50^*^
	Mucification, goblet, bronchial, focal	1.3 ± 0.96	3.7 ± 0.50^#^	3.5 ± 0.58	3.3 ± 0.50	1.3 ± 0.96^*^
Trachea	Inflammation, submucosal, focal	1.5 ± 0.58	4.0 ± 0.82^#^	3.8 ± 0.50	3.8 ± 0.96	2.3 ± 0.50^*^

^1^Degree of lesions was graded from one to five depending on severity: 1 = minimal (<1%); 2 = slight (1%–25%); 3 = moderate (26%–50%); 4 = moderate/severe (51%–75%); 5 = severe/high (76%–100%). Data are expressed as mean ± standard error of the mean (SEM), with n = 4 mice per group.

^#^ P < 0.05 compared to the unsensitized control.

^*^ P < 0.05 compared to the sensitized Der p.

### 3.2 Effects of DATS, DADS, and SAC on the release of cytokines by macrophages in BALF of Der p-induced allergic asthmatic mice

**FIGURE 2:**
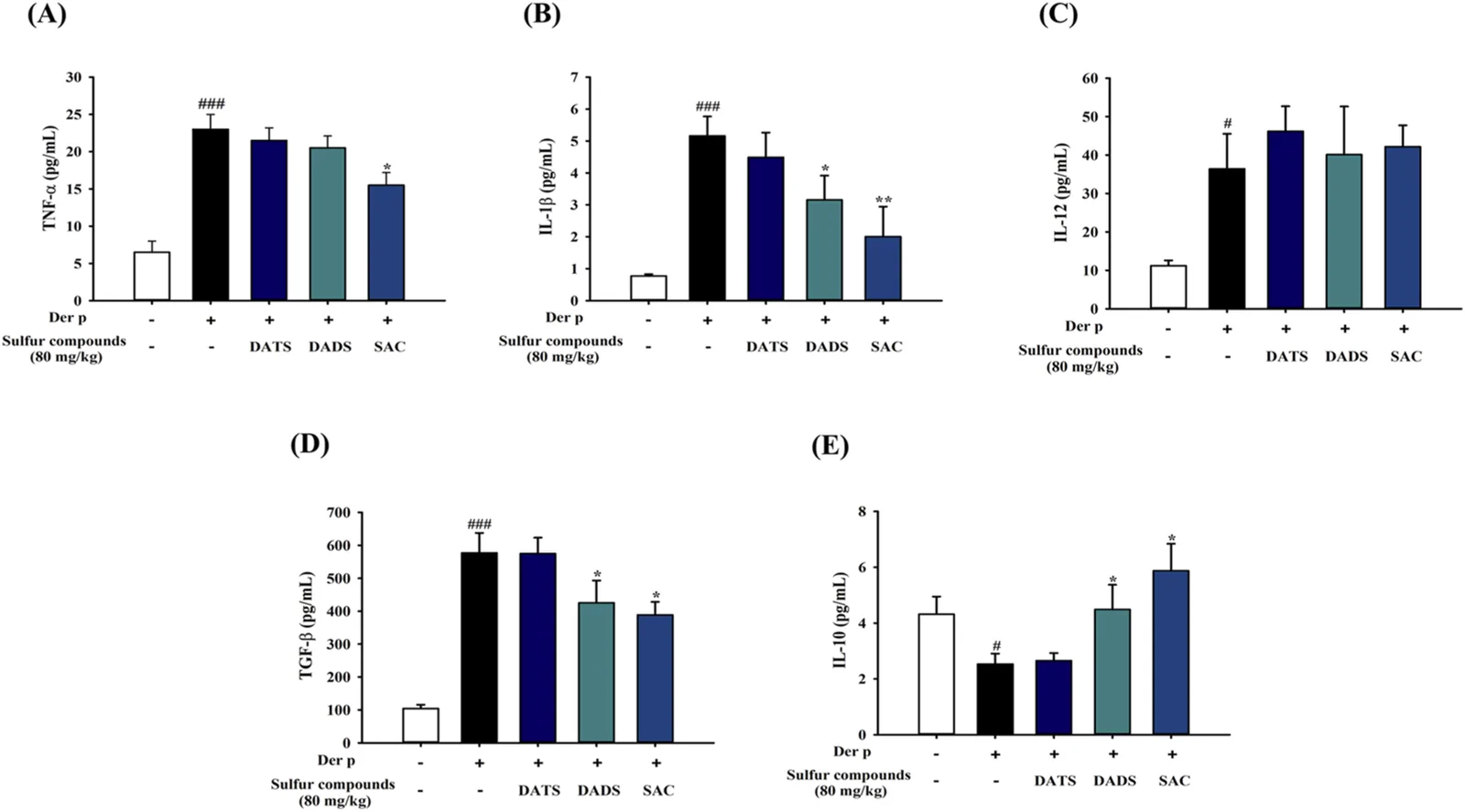
Effects of diallyl disulfide (DADS), diallyl trisulfide (DATS), and S-allyl cysteine (SAC) on the release of cytokines by macrophages in bronchoalveolar lavage fluid (BALF). DATS, DADS, and SAC were administered at a dosage of 80 mg/kg. Panels show concentrations of TNF-α **(A)**, IL-1β **(B)**, IL-12 **(C)**, TGF-β **(D)**, and IL-10 **(E)** in BALF from unsensitized controls, sensitized controls, and sensitized mice treated with DATS, DADS, or SAC. Cytokine concentrations were measured using ELISA kits according to the manufacturer’s instructions. Data are expressed as mean ± SEM (n = 8). ^#^ *P* < 0.05, ^###^ *P* < 0.001 compared to the unsensitized control; ^*^ *P* < 0.05, ^**^ *P* < 0.01 compared to the sensitized control.

Currently, it is well established that respiratory tract inflammation, particularly when triggered by infections or irritants, increases bronchial reactivity, rendering the airways more sensitive to constriction. Macrophages, key regulators of immune responses, secrete both pro- and anti-inflammatory cytokines upon activation. In response to inflammatory stimuli, they release pro-inflammatory mediators such as TNF-α, IL-1, and IL-12, which initiate and amplify the inflammatory cascade by recruiting additional immune cells to the site of infection or injury and promoting pathogen clearance. Conversely, they can produce anti-inflammatory cytokines such as IL-10, which attenuate the inflammatory response and help prevent excessive tissue damage (Hammad and Lambrecht, 2021). As shown in Figure 2, compared with the unsensitized control group, the Der p group showed a significant increase in the levels of pro-inflammatory mediators (TNF-α, IL-1β, and IL-12) and the anti-inflammatory cytokine TGF-β in BALF. In contrast, the anti-inflammatory cytokine IL-10 was significantly decreased. It was observed that DATS had no significant effect on these Der p-induced changes. Compared with the Der p group, DADS partially reversed the Der p-induced alterations in IL-1β (from 5.16 ± 0.61 to 3.16 ± 0.76 pg/mL, *P* < 0.05), TGF-β (from 577.01 ± 60.71 to 425.30 ± 67.90 pg/mL *P* < 0.05), and IL-10 (from 2.53 ± 0.38 to 4.49 ± 0.88 pg/mL *P* < 0.05), whereas IL-12 and TNF-α remained unaffected. SAC reduced TNF-α from 23.00 ± 2.00 to 15.50 ± 1.70 pg/mL (*P* < 0.05), IL-1β from 5.16 ± 0.61 to 2.00 ± 0.94 pg/mL (*P* < 0.01), and TGF-β from 577.01 ± 60.71 to 388.75 ± 39.50 pg/mL (*P* < 0.05), while increasing IL-10 from 2.53 ± 0.38 to 5.88 ± 0.97 pg/mL (*P* < 0.05). Except for IL-12, SAC significantly attenuated the Der p-induced cytokine alterations, indicating that it exerted the strongest regulatory effect among the three garlic-derived allyl sulfides.

### 3.3 Effects of DATS, DADS, and SAC on the release of cytokines by Th1/Th2 cells in BALF of Der p-induced allergic asthmatic mice

**FIGURE 3:**
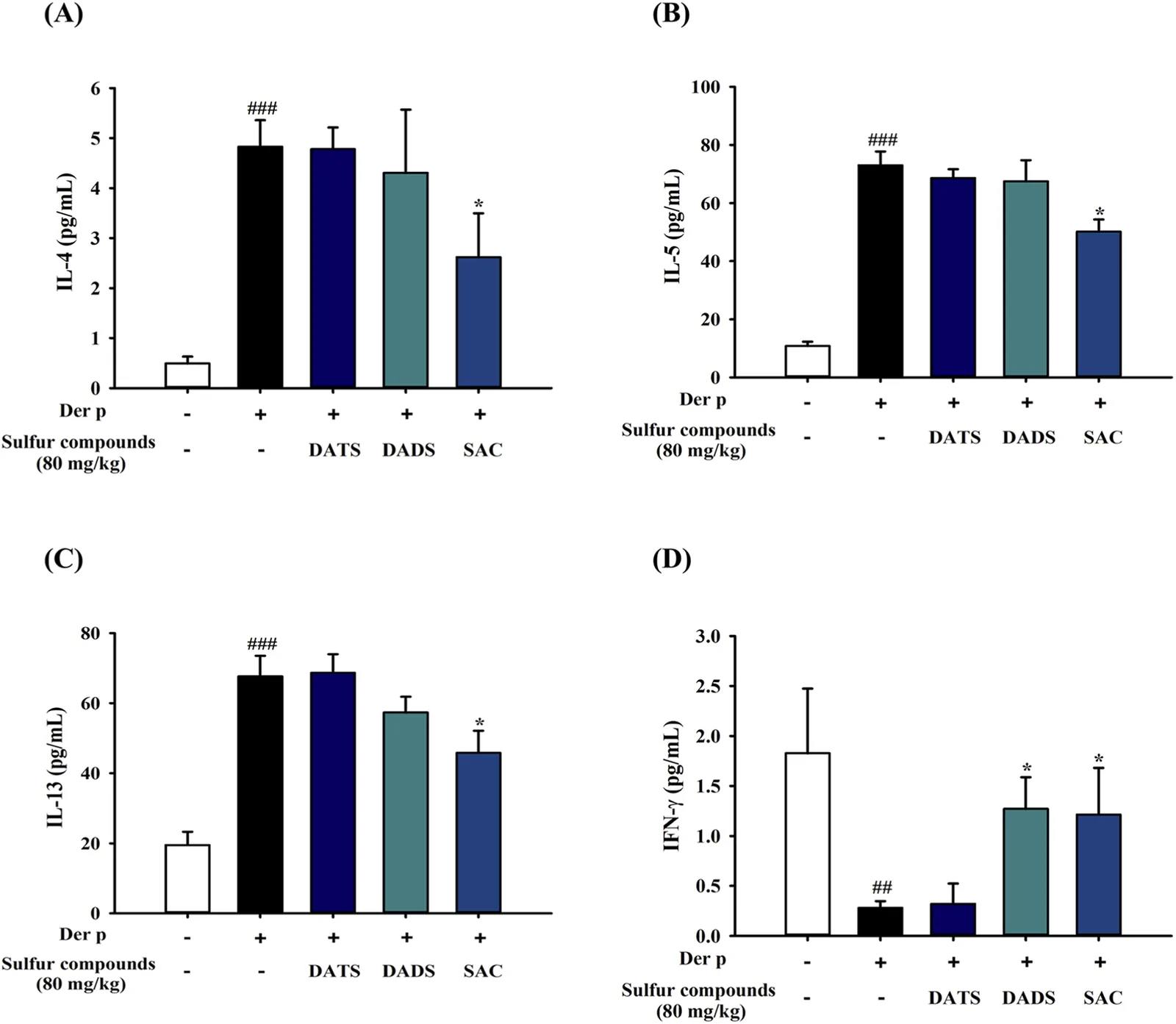
Effects of diallyl disulfide (DADS), diallyl trisulfide (DATS), and S-allyl cysteine (SAC) on the release of cytokines by Th1/Th2 cells in bronchoalveolar lavage fluid (BALF). DADS, DATS, and SAC were administered at a dosage of 80 mg/kg. Panels show concentrations of IL-4 **(A)**, IL-5 **(B)**, IL-13 **(C)**, and IFN-γ **(D)** in BALF from unsensitized controls, sensitized controls, and sensitized mice treated with DATS, DADS, or SAC. Cytokine concentrations were measured using ELISA kits according to the manufacturer’s instructions. Data are expressed as mean ± SEM (n = 8). ^##^ *P* < 0.01, ^###^ *P* < 0.001 compared to the unsensitized control; ^*^ *P* < 0.05 compared to the sensitized control.

When the body is exposed to Der p, a major house dust mite allergen, antigen-presenting cells (such as dendritic cells) engulf and process it into small fragments. These fragments are then presented to T helper cells. In allergic individuals, these T helper cells differentiate into Th2 cells, which secrete various cytokines. A key cytokine in this process is IL-4. Furthermore, macrophages polarize into M1 or M2 phenotypes, promoting Th1 or Th2 responses, respectively. Th1 cytokines like IFN-γ drive M1 polarization and cellular immunity, whereas Th2 cytokines such as IL-4, IL-5, and IL-13 induce M2 polarization and may suppress Th1 responses (Barnes, 2001). Therefore, this study evaluated the effects of DATS, DADS, and SAC on the release of cytokines by Th1/Th2 cells in the BALF of Der p-induced allergic asthmatic mice (Figure 3). Compared with the unsensitized control group, the Der p group exhibited a significant decrease in IFN-γ level in BALF, while IL-4, IL-5, and IL-13 levels were significantly increased. In sensitized mice, DATS did not significantly affect these alterations. DADS partially reversed the decrease in IFN-γ from 0.28 ± 0.07 to 1.27 ± 0.38 pg/mL (*P* < 0.05) but did not affect IL-4, IL-5, or IL-13. Compared with the Der p group, SAC increased IFN-γ from 0.28 ± 0.07 to 1.21 ± 0.47 pg/mL and reduced IL-4 from 4.83 ± 0.53 to 2.62 ± 0.88 pg/mL, IL-5 from 73.00 ± 4.73 to 50.17 ± 4.17 pg/mL, and IL-13 from 67.67 ± 5.85 to 45.83 ± 6.31 pg/mL (all *P* < 0.05; Figure 3). Similarly, SAC exhibited the most comprehensive regulation of Th1/Th2 cytokines, while DADS only affected IFN-γ, and DATS had virtually no effect.

### 3.4 Effects of DATS, DADS, and SAC on the IgE, IgG1, and IgG2a in serum of Der p-induced allergic asthmatic mice

IL-4 is essential for inducing IgG1 and IgE class switching in B cells, resulting in IgG1 and IgE production by plasma cells, a key trigger of allergic reactions (Cernescu et al., 2021).

**FIGURE 4:**
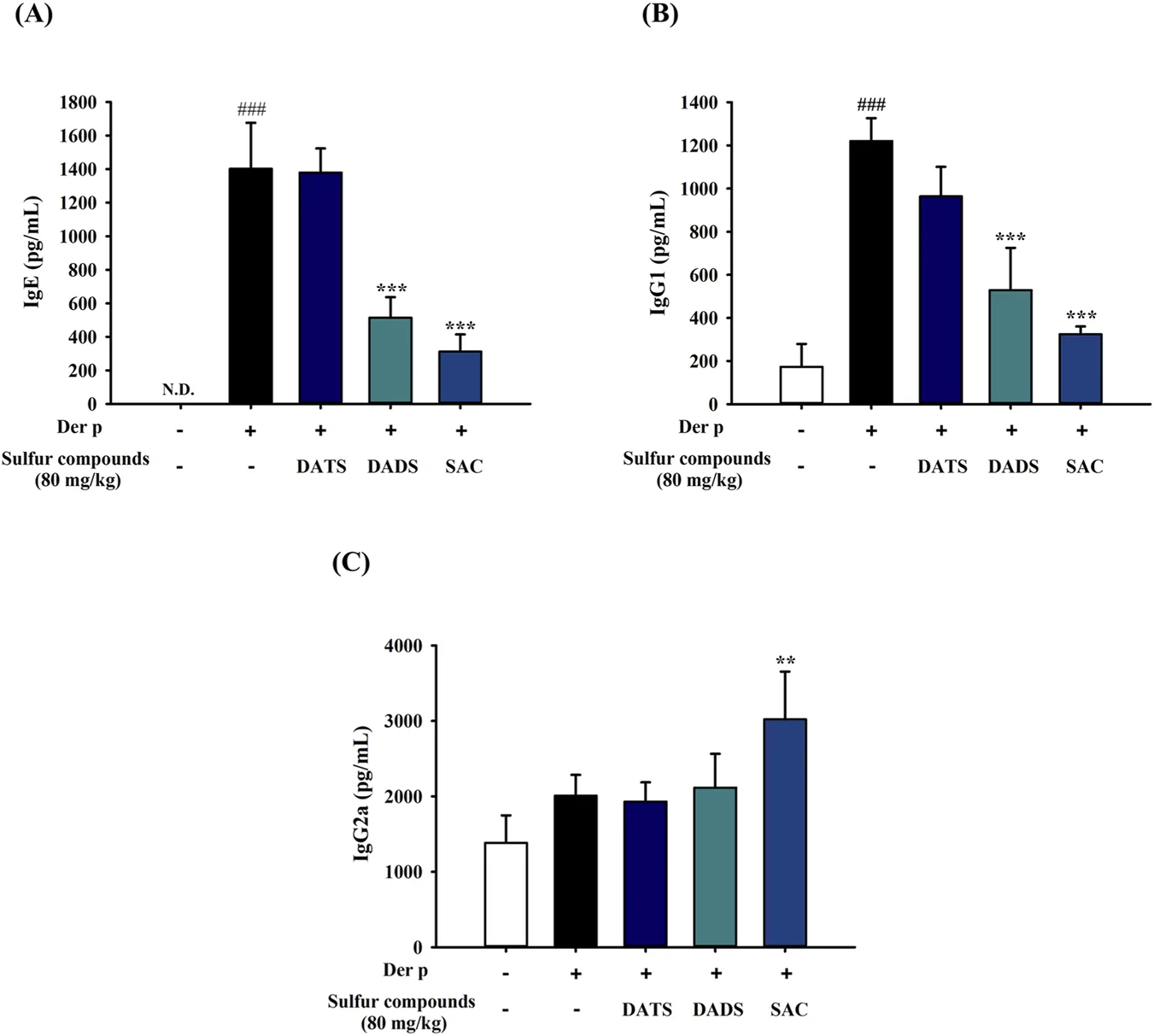
Effects of diallyl disulfide (DADS), diallyl trisulfide (DATS), and S-allyl cysteine (SAC) on the IgE **(A)**, IgG1 **(B)**, and IgG2a **(C)** in serum of Der p-induced allergic asthmatic mice. DATS, DADS, and SAC were administered at a dosage of 80 mg/kg. Panels show concentrations of IgE, IgG1, and IgG2a in serum from unsensitized controls, sensitized controls, and sensitized mice treated with DATS, DADS, or SAC. IgE, IgG1, and IgG2a concentrations were measured using ELISA kits according to the manufacturer’s instructions. Data are expressed as mean ± SEM (n = 8). ^###^ *P* < 0.001 compared to the unsensitized control; ^**^ *P* < 0.01, ^***^ *P* < 0.001 compared to the sensitized control.

According to the above results, the Der p group exhibited a significant increase in IL-4 levels in BALF (Figure 3). To further elucidate whether Der p-induced IL-4 promotes B-cell class switching and plasma cell differentiation leading to IgE production, serum immunoglobulin levels were evaluated. As shown in Figure 4, serum IgE levels were significantly elevated in sensitized mice compared with normal controls. IgG1 is produced early in the immune response, whereas IgE is mainly associated with allergic reactions, arising after initial allergen exposure and persisting through sensitization. The amount of Der p-specific IgG1 in the serum of sensitized mice was also significantly higher than that in normal mice. This study also assessed the effects of DATS, DADS, and SAC on serum IgG1 and IgE levels in Der p-induced allergic asthmatic mice. Compared with the Der p group, DATS had no significant effect. DADS reduced IgE from 1402.19 ± 273.75 to 513.83 ± 122.27 pg/mL and IgG1 from 1220.50 ± 105.77 to 529.00 ± 196.43 pg/mL (all *P* < 0.001). Also, SAC reduced serum IgE from 1402.19 ± 273.75 to 311.80 ± 102.75 pg/mL and IgG1 from 1220.50 ± 105.77 to 324.33 ± 36.02 pg/mL. Both SAC and DADS treatments significantly reduced IgE and IgG1 levels compared with sensitized controls. In addition, serum Der p-specific IgG2a was significantly elevated only in the SAC-treated group (from 2009.33 ± 275.63 to 3,022.00 ± 632.39 pg/mL, *P* < 0.01) relative to the Der p group. IgG2a requires IFN-γ (Th1-type cytokine) for switching and tends to appear later, usually in more chronic or Th1-skewed responses. These results are consistent with the finding that SAC treatment significantly increased IFN-γ levels in BALF.

### 3.5 Effects of DATS, DADS and SAC on the PI3K and NF-κB protein expressions in lung tissues of Der p-induced allergic asthmatic mice

**FIGURE 5:**
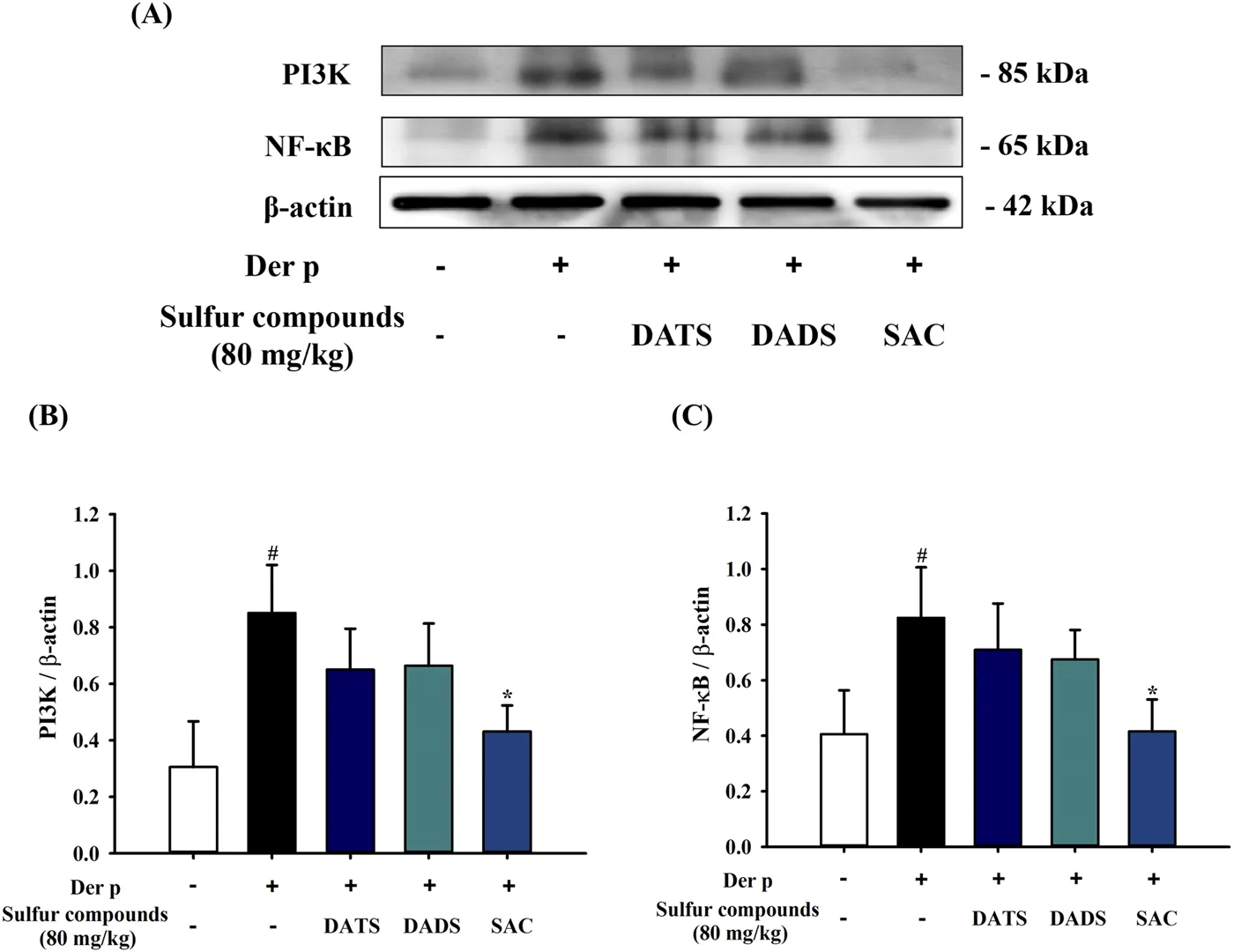
Effects of diallyl disulfide (DADS), diallyl trisulfide (DATS), and S-allyl cysteine (SAC) on the PI3K and NF-κB protein expressions in lung tissues of Der p-induced allergic asthmatic mice. **(A)** Representative Western blot images of PI3K, NF-κB, and β-actin. **(B,C)** Quantitative analysis of PI3K/β-actin and NF-κ/β-actin. The effects of DADS, DATS, and SAC on the expression of PI3K and NF-κB proteins were detected by Western blot analysis. DADS, DATS, and SAC were administered at a dosage of 80 mg/kg. The degree of PI3K and NF-κB protein expression was quantified by Lane 1D Gel imaging analysis software. Data are expressed as mean ± SEM (n = 8). ^#^ *P* < 0.05 compared to the unsensitized control; ^*^ *P* < 0.05 compared to the sensitized control.

PI3K and NF-κB are key signaling pathways involved in the pathogenesis of asthma, contributing to inflammatory responses, airway remodeling, and immune cell activation (Rao et al., 2017). Therefore, this study investigated whether PI3K and NF-κB are involved in Der p-induced allergic asthma in mice. As shown in Figure 5, Der p sensitization significantly increased the protein expression of PI3K and NF-κB in the lung tissues compared with the unsensitized control group. SAC significantly inhibited the Der p-induced upregulation of PI3K (*P* < 0.05) and NF-κB (*P* < 0.05). However, DATS and DADS had no significant effect on PI3K and NF-κB protein levels induced by Der p. Among the three garlic-derived allyl sulfides, SAC exhibited the strongest inhibitory effect on PI3K and NF-κB protein expression in lung tissues (Figure 5).

### 3.6 Effect of SAC on A549 cell viability following Der p exposure

**FIGURE 6:**
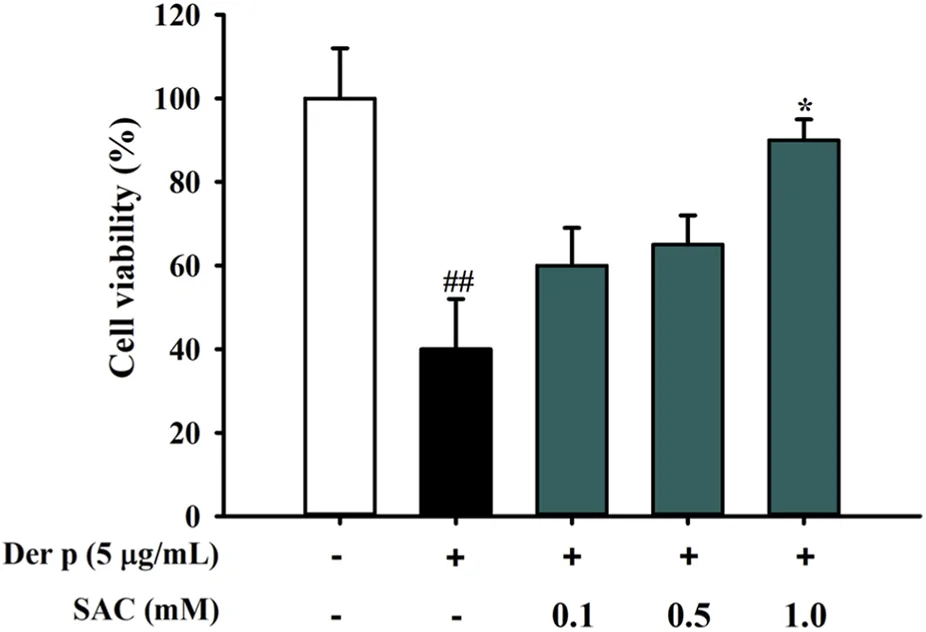
Effect of S-allyl cysteine (SAC) on A549 cell viability following Der p exposure. A549 cells were treated with Der p (5 μg/mL) in the absence or presence of SAC (0.1, 0.5, or 1 mM) for 24 h, and cell viability was determined by MTT assay. Data are expressed as mean ± SEM (n = 3). ^##^ *P* < 0.01 compared to the unsensitized control; ^*^ *P* < 0.05 compared to the sensitized control.

A549 cells are widely used to model epithelial responses to allergens and allow for reproducible assessment of intracellular signaling pathways, such as PI3K/NF-κB activation, under controlled conditions (Adam et al., 2006). In the *in vivo* experiments of this study, Der p has been shown to induce epithelial inflammation in mouse models, while SAC alleviates these effects. Building on these findings, this study investigated whether Der p induces cell death in A549 cells and whether SAC can mitigate this effect. Exposure to Der p (5 μg/mL) for 24 h markedly reduced A549 cell viability to approximately 40% of control levels (*P* < 0.001). Treatment with SAC attenuated this cytotoxic effect in a concentration-dependent manner, with 1 mM SAC significantly restoring cell viability to nearly control levels (*P* < 0.05 vs. Der p alone). Lower concentrations of SAC (0.1 and 0.5 mM) partially improved viability, although the differences were not statistically significant (Figure 6). Our *in vitro* findings demonstrated that SAC mitigates Der p-induced cytotoxicity in A549 cells, suggesting its potential to preserve epithelial integrity under allergen challenge. While this effect does not directly confirm protection against airway injury *in vivo*, our murine model data showing reduced epithelial remodeling and inflammation provide supportive evidence that SAC may alleviate structural damage associated with chronic allergic asthma.

### 3.7 Effects of DATS, DADS, and SAC on PI3K/Akt/NF-κB signaling in Der p-stimulated A549 cells

**FIGURE 7:**
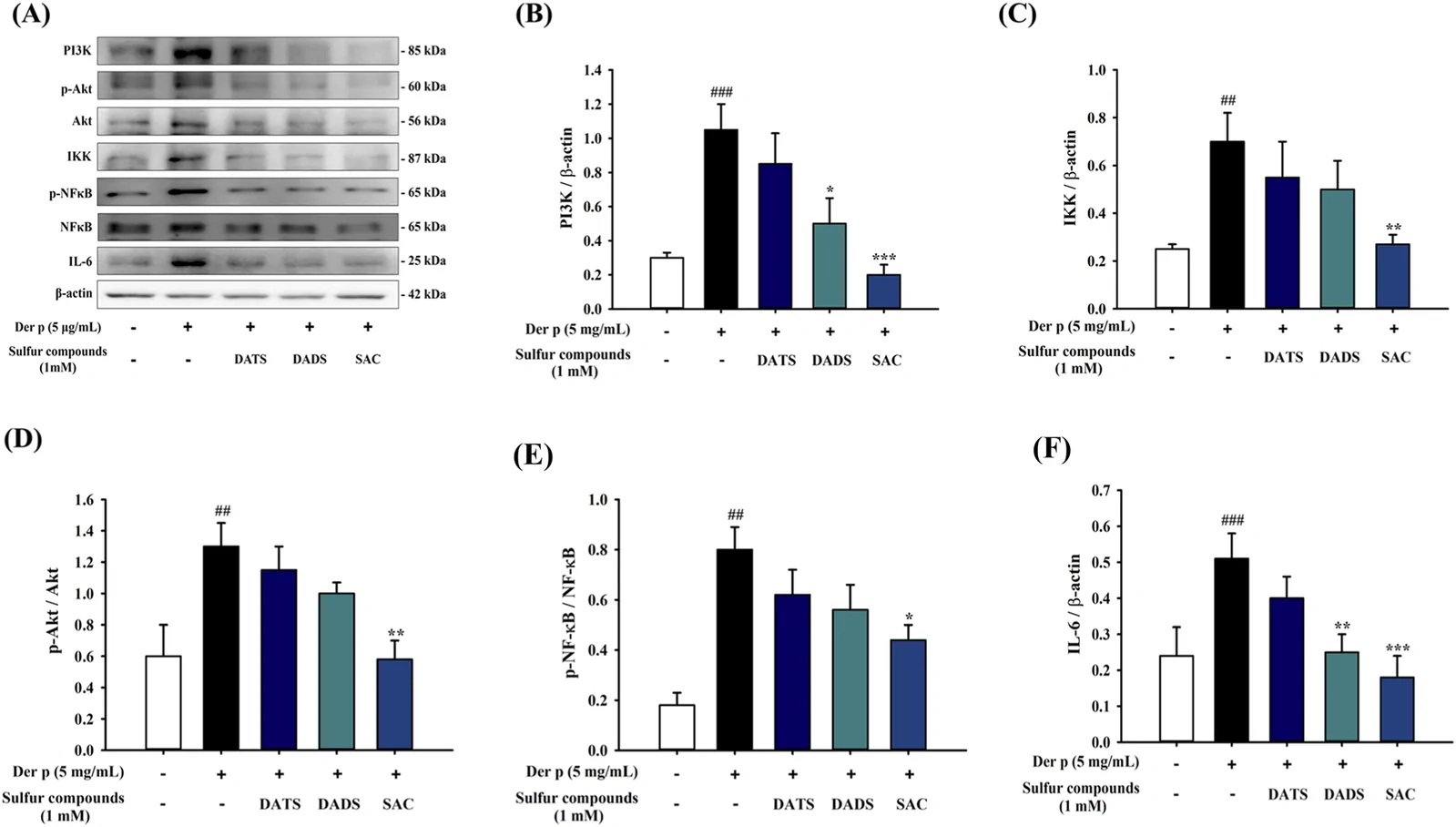
Effects of diallyl disulfide (DADS), diallyl trisulfide (DATS), and S-allyl cysteine (SAC) on PI3K/Akt/NF-κB signaling and IL-6 expression in Der p-stimulated A549 cells. **(A)** Representative Western blot images of PI3K, p-Akt, Akt, IKK, p-NF-κB, NF-κB, IL-6, and β-actin. **(B–F)** Quantitative analysis of PI3K/β-actin, IKK/β-actin, p-Akt/Akt, p-NF-κB/NF-κB, and IL-6/β-actin. A549 cells were treated with Der p (5 μg/mL) in the presence or absence of DATS, DADS, or SAC (1 mM). Western blot analysis of PI3K/Akt/NF-κB signaling and IL-6 in A549 cells following Der p stimulation and treatment with garlic-derived compounds. Protein expression levels of PI3K, IKK, p-Akt, Akt, p-NF-κB, NF-κB, IL-6, and β-actin (loading control) were quantified by Lane 1D Gel imaging analysis software. Data are expressed as mean ± SEM (n = 3). ^##^ *P* < 0.01, ^###^ *P* < 0.001 compared to the unsensitized control; ^*^ *P* < 0.05, ^**^ *P* < 0.01, ^***^ *P* < 0.001 compared to the sensitized control.

In this study, A549 cells served as a simplified model to examine Der p-induced activation of the PI3K/NF-κB signaling pathway in the airway epithelium. The PI3K/Akt pathway contributes to NF-κB activation through the phosphorylation of IκB kinase (IKK), leading to IκBα degradation and subsequent NF-κB nuclear translocation. In addition to evaluating PI3K and NF-κB protein expression, we also detected the protein expression of Akt, p-Akt, IKK, and p-NF-κB. Exposure to Der p significantly increased the expression of PI3K and IKK, as well as the phosphorylation of Akt and NF-κB. These results indicate that Der p activates the PI3K/Akt/IKK signaling pathway, leading to the activation of NF-κB and subsequent airway inflammation. Consistent with the *in vivo* findings, SAC most effectively suppressed PI3K/Akt/IKK/NF-κB signaling, followed by DADS, while DATS had minimal effects. SAC significantly reduced the phosphorylation of Akt (*P* < 0.01) and NF-κB (*P* < 0.05) and the expression of PI3K (*P* < 0.001), IKK (*P* < 0.01), and IL-6 (*P* < 0.001) in Der p-stimulated A549 cells. DADS also showed significant reductions in PI3K and IL-6 (Figure 7). In conclusion, these findings collectively indicated that SAC effectively mitigates Der p-induced airway inflammation by downregulating the PI3K/Akt/IKK signaling pathway and suppressing the activation of NF-κB.

### 3.8 Molecular docking analysis of garlic-derived compounds with NF-κB

**FIGURE 8:**
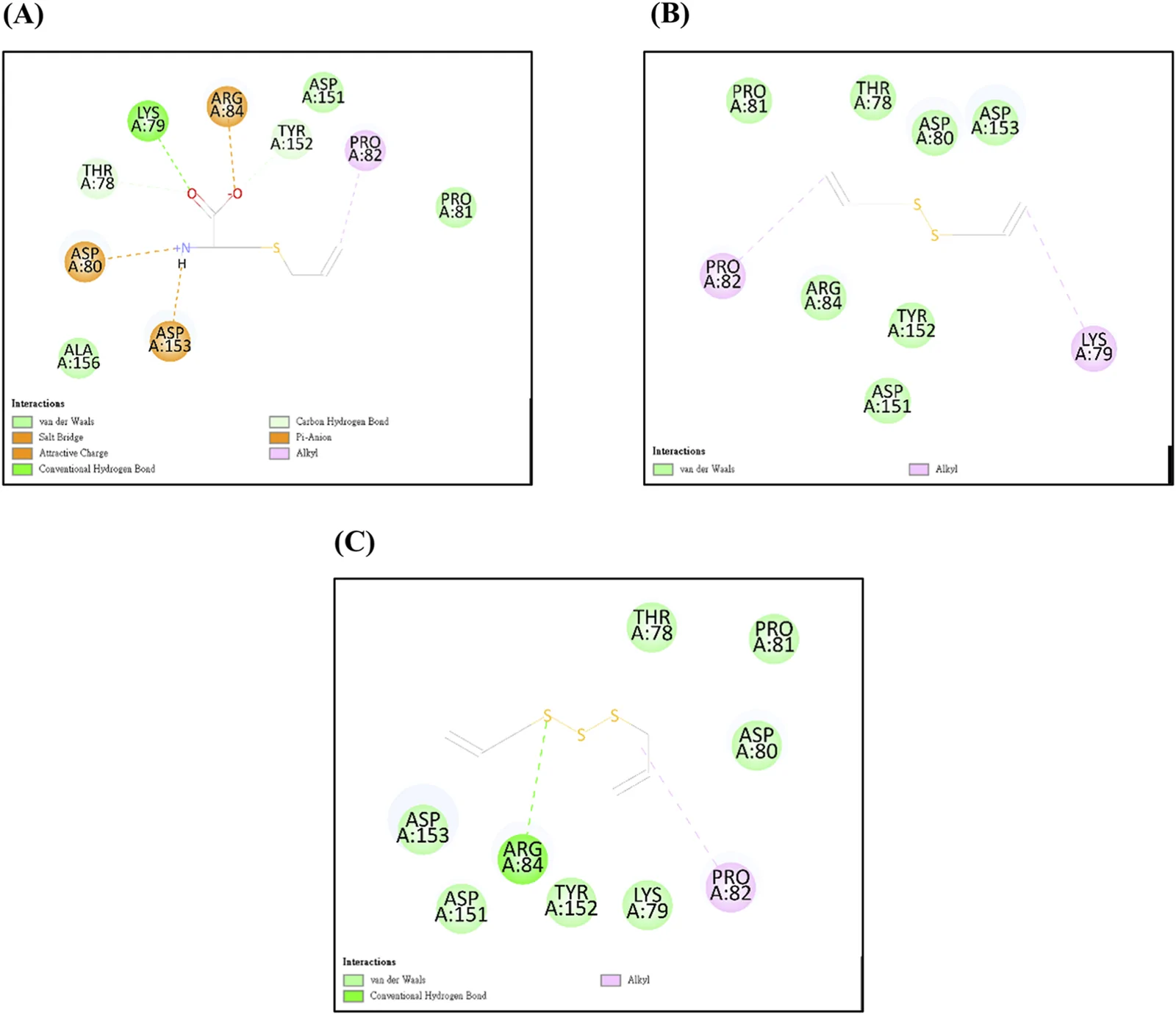
Molecular Docking Analysis of Garlic-Derived Compounds with NF-κB. The 2 D protein-ligand interaction map derived from molecular docking of NF-κB protein (PDB ID: 2RAM) with the *S*-allyl cysteine (SAC), diallyl disulfide (DADS), and diallyl trisulfide (DATS). **(A)** Docking interaction diagram of SAC with 2RAM. SAC forms hydrogen bonds with Lys79, a salt bridge with Asp80 and Asp153, an attractive charge interaction with Arg84, and hydrophobic interactions with Pro82. **(B)** Docking interaction diagram of diallyl disulfide (DADS) with 2RAM. DADS interacts mainly through alkyl contacts with Lys79 and Pro82, supported by van der Waals interactions with surrounding residues. **(C)** Docking interaction diagram of diallyl trisulfide (DATS) with 2RAM. DATS forms a hydrogen bond with Arg84 and hydrophobic interactions with Pro82, but shows fewer stabilizing interactions compared to SAC.

Molecular docking was used as a predictive computational tool for structural visualization and to illustrate the potential binding mode of garlic-derived compounds within the functional domain of NF-κB p65. We evaluated the predicted binding affinities of three allyl sulfides, *S*-allyl cysteine (SAC), diallyl disulfide (DADS), and diallyl trisulfide (DATS), with the NF-κB p65 homodimer (PDB ID: 2RAM). The strength of these interactions was measured by their (−)CDOCKER interaction energies, where a higher score indicates stronger binding. As shown in our results, SAC demonstrated the highest docking score (32.038), followed by DADS (16.0452) and DATS (14.7744), suggesting the highest predicted binding affinity among the three compounds. The docking model predicted that SAC involved multiple non-covalent interactions. The carboxyl group of SAC formed a conventional hydrogen bond with Lys79 (Figure 8A). Additionally, the carboxyl group engaged in two carbon-hydrogen bonds with Thr78 and Tyr152, further anchoring the molecule (Figure 8A). The amino group of SAC formed a salt bridge with two negatively charged amino acid residues, Asp80 and Asp153, while the carboxyl group of SAC formed an attractive charge interaction with Arg84. Meanwhile, the hydrophobic allyl group of SAC is also involved in an alkyl interaction with Pro82. The complex is further stabilized by van der Waals forces with surrounding residues like Ala156, Pro81, and Asp151 (Figure 8A). In contrast, DADS primarily engaged in alkyl interactions with Lys79 and Pro82, supported by van der Waals contacts (Figure 8B). DATS exhibited the weakest binding affinity, forming a single hydrogen bond with Arg84 and some hydrophobic interactions with Pro82, but showed fewer predicted electrostatic interactions than SAC (Figure 8C). The docking simulation provided a qualitative structural model illustrating potential polar and electrostatic interactions (Lys79, Asp80, Asp153) between SAC and the NF-κB p65 active domain, aligning with our *in vitro* pathway findings.

## 4 Discussion

This study explored the protective effects of allyl sulfides derived from garlic on a Der p-induced allergic asthmatic animal model, focusing specifically on their immunomodulatory and anti-inflammatory mechanisms. In addition to animal models, we also investigated the effects of allyl sulfide on the Der p-induced PI3K/Akt/NF-κB pathway in A549 cells. We anticipate that these allyl sulfides may offer potential as natural alternatives or adjunctive therapies for asthma. Histopathological examination and semi-quantitative scoring revealed that Der p exposure induced airway inflammation and structural alterations, including epithelial thickening and inflammatory cell infiltration. These changes were significantly attenuated in the SAC-treated group. However, DADS showed partial improvement, and DATS demonstrated moderate effects. The semi-quantitative histological scores were consistent with these observations, supporting the conclusion that SAC exerted the most consistent protective effect among the tested compounds in this model.

Macrophages are key regulators of the immune response, playing a crucial role in both initiating and resolving inflammation. Initially, macrophages adopt a pro-inflammatory M1 phenotype, releasing cytokines such as TNF-α, IL-1β, IL-12, and IL-23 to combat inflammatory stimuli. While essential for clearing pathogens, a prolonged M1 response can lead to tissue damage. To prevent this, macrophages can shift to an anti-inflammatory M2 phenotype. M2 macrophages secrete high levels of cytokines like IL-10 and TGF-β, which suppress the inflammatory cascade, promote tissue repair, and restore homeostasis. This transition is vital for the resolution of inflammation and is often dysregulated in chronic inflammatory diseases like asthma, rheumatoid arthritis, and inflammatory bowel disease (Wang et al., 2014; Zhu et al., 2014; Cutolo et al., 2022). The study investigated the effects of garlic-derived compounds on pro- and anti-inflammatory cytokine profiles in Der p-induced allergic asthma. Consistent with the role of macrophages in inflammation, the Der p group showed a significant increase in pro-inflammatory cytokines (TNF-α, IL-1β, IL-12) alongside an increase in the pro-fibrotic cytokine TGF-β and a notable decrease in the anti-inflammatory cytokine IL-10 in the BALF. Treatment with the garlic compounds yielded different outcomes. DATS had no significant effect on the levels of either pro- or anti-inflammatory mediators. DADS partially mitigated some of the Der p-induced changes, specifically reversing the levels of IL-1β, IL-10, and TGF-β, but had no impact on IL-12 or TNF-α. In contrast, SAC demonstrated the most comprehensive effect, significantly reversing the Der p-induced changes in TNF-α, IL-1β, IL-10, and TGF-β. This suggests that SAC modulates the balance of inflammatory cytokines more effectively than DADS or DATS, contributing to the resolution of inflammation and tissue repair.

Macrophages can be polarized into M1 or M2 phenotypes, which in turn can promote or suppress Th1 and Th2 cell responses, respectively. The Th1/Th2 cytokine imbalance is a hallmark of allergic asthma, characterized by reduced IFN-γ and elevated Th2-associated cytokines such as IL-4, IL-5, and IL-13 (Barnes, 2001). This study investigated the impact of Der p and subsequent treatment with garlic compounds on the Th1/Th2 cytokine balance in allergic asthma. The results showed that Der p exposure significantly skewed this balance, leading to a notable decrease in the Th1 cytokine IFN-γ and a significant increase in the Th2 cytokine IL-4 in the BALF. This imbalanced Th1/Th2 profile is characteristic of allergic responses (Barnes, 2001; Truyen et al., 2006; Durrant and Metzger, 2010). The garlic compounds had varying effects on this cytokine imbalance. DATS demonstrated no significant effect on either IFN-γ or IL-4 levels. DADS partially restored the Th1/Th2 balance by increasing IFN-γ levels, but it failed to alter the elevated IL-4 significantly. Most importantly, SAC was the only compound to significantly correct both the decrease in IFN-γ and the increase in IL-4. These suggest that SAC effectively modulates the Th1/Th2 response, helping to re-establish a more balanced immune profile. This ability to restore the Th1/Th2 balance likely contributes to SAC’s potent therapeutic effects against Der p-induced allergic asthma.

Exposure to Der p was shown to induce a hallmark allergic response, characterized by elevated levels of the Th2 cytokines IL-4 and IL-13, which are critical for B cell class switching. This was further confirmed by the significant increase in both total serum IgE and Der p-specific IgG1 in sensitized mice, indicating a robust allergic humoral response. The effectiveness of the garlic compounds in mitigating this response varied. DATS had no significant effect on either IgE or IgG1 levels. In contrast, both DADS and SAC significantly reduced serum IgE and IgG1. Remarkably, SAC uniquely increased serum levels of Der p-specific IgG2a. This is a particularly important finding, as IgG2a production is dependent on the Th1 cytokine IFN-γ. Since our results also confirmed that SAC treatment significantly increased IFN-γ levels, this suggests that SAC not only suppresses the allergic Th2-mediated response but also actively promotes a protective Th1-mediated immune response. This dual mechanism of action, downregulating allergic antibodies (IgE and IgG1) while simultaneously upregulating a Th1-associated antibody (IgG2a), highlights SAC’s superior ability to modulate the immune system toward a non-allergic, protective state.

PI3K signaling promotes the recruitment and activation of immune cells, including eosinophils, neutrophils, macrophages, and dendritic cells, thereby perpetuating airway inflammation (Lee et al., 2006; Katholnig et al., 2013; Datler et al., 2019; Song et al., 2025). Another critical signaling mediator in asthma is NF-κB, a transcription factor that is involved in the expression of numerous pro-inflammatory genes, including cytokines (IL-4, IL-5, IL-6, TNF-α), chemokines (eotaxin, MCP-1), and adhesion molecules (ICAM-1) (Moynagh, 2005; Park et al., 2022; Kang et al., 2025; Moneva-Sakelarieva et al., 2025). This study investigated the role of the PI3K/NF-κB signaling pathway in Der p-induced allergic asthma. Western blot analysis confirmed that Der p exposure significantly upregulated the protein expression of both PI3K and NF-κB in the lung tissues of asthmatic mice, highlighting their involvement in the pathogenesis of airway inflammation and remodeling. Treatment with garlic-derived compounds demonstrated varying efficacy in targeting these pathways. DATS and DADS had no significant effect on PI3K and NF-κB expression. In contrast, SAC proved to be a highly effective inhibitor, as it significantly suppressed the Der p-induced upregulation of both PI3K and NF-κB protein levels. These findings provide molecular evidence that the therapeutic effects of SAC are associated, at least in part, with modulation of key inflammatory signaling pathways, as reflected by reduced expression of PI3K and NF-κB.

A549 cells were used as an *in vitro* model to examine early epithelial signaling events triggered by Der p exposure. Although A549 cells do not fully reproduce the complex airway remodeling observed *in vivo*, they are widely applied to investigate allergen-induced intracellular signaling and inflammatory mediator release under controlled conditions (Asokananthan et al., 2002; Trifilieff et al., 2003; Adam et al., 2006). In this study, Der p stimulation markedly increased PI3K and IKK expression, as well as phosphorylation of Akt and NF-κB in A549 cells, indicating activation of the PI3K/Akt/NF-κB pathway. These findings are consistent with previous reports demonstrating that allergen-induced PI3K/Akt/NF-κB pathway activation is central to the pathogenesis of asthma (Park et al., 2022; Yan et al., 2024; Kang et al., 2025; Song et al., 2025). Interestingly, treatment with garlic-derived organosulfur compounds (DATS, DADS, and SAC) attenuated these effects, with SAC exerting the most pronounced inhibition. Specifically, SAC reduced PI3K and IKK expression and decreased the phosphorylation of Akt and NF-κB, suggesting that its anti-inflammatory effects are associated with suppression of this signaling cascade. Taken together, these results suggest that SAC mitigates Der p-induced airway inflammation, which is accompanied by reduced PI3K/Akt/IKK/NF-κB signaling. This mechanistic insight provides a potential therapeutic strategy for controlling allergic airway inflammation through dietary sulfur-containing compounds.

SAC, a water-soluble compound found in garlic, has shown the ability to improve respiratory function and mitigate allergic inflammatory responses. Our previous research identified SAC as a potent agent against allergic asthma (Hsieh et al., 2019). Known for its anti-inflammatory properties, SAC is considered a significant contributor to garlic’s pharmacological activities (Arreola et al., 2015; Johnson et al., 2018). The current study further supports these earlier findings, demonstrating that the water-soluble SAC component is highly effective, suggesting it is a key active ingredient in garlic for combating allergic asthma. Moreover, SAC exerted dual effects, reducing IgE and IgG1 while elevating IgG2a, indicating its strong capacity to redirect immunity toward a non-allergic, protective state. In this study, SAC demonstrated therapeutic potential in allergic asthma by preserving epithelial integrity and attenuating airway inflammation associated with reduced PI3K/NF-κB signaling. Histopathological evidence revealed that SAC treatment effectively maintained the structural integrity of bronchi and alveoli, with nearly normal epithelial architecture and minimal inflammatory infiltration. Consistent with these *in vivo* observations, *in vitro* experiments using A549 alveolar epithelial cells showed that SAC markedly improved cell viability following Der p exposure and significantly suppressed PI3K/NF-κB activation, a key pathway mediating allergen-induced inflammation. Collectively, these findings indicate that SAC mitigates allergen-induced epithelial damage and structural remodeling, supporting its potential as a promising candidate for the prevention and management of allergic asthma. In addition, SAC belongs to the family of cysteine-derived compounds. N-acetylcysteine (NAC), a clinically used mucolytic and antioxidant agent, is another member of this group. Previous studies have reported that NAC ameliorates respiratory allergic inflammation (Lee et al., 2020). In the present study, SAC also exhibited protective effects against respiratory inflammation. These findings support further investigation of cysteine-derived compounds for respiratory inflammation, and future studies may also explore their structure-activity relationships (SAR). However, the present study did not include a direct comparison with established anti-asthmatic therapies, such as corticosteroids or leukotriene receptor antagonists. Future comparative studies with established anti-asthmatic therapies will help determine the relative efficacy of SAC.

Furthermore, our docking study explored the potential interactions between garlic-derived allyl sulfides and the NF-κB p65 homodimer (PDB ID: 2RAM). Among the compounds tested, SAC displayed the highest predicted binding affinity for NF-κB, outperforming both DADS and DATS. The docking model predicted that SAC formed more electrostatic and hydrogen-bonding interactions than DADS and DATS. These findings were consistent with our experimental observations, which showed that SAC exerted the strongest anti-inflammatory effects and the greatest reduction in PI3K/NF-κB signaling in both *in vivo* and *in vitro* models. However, the docking analysis was used only to evaluate potential compound–protein interactions and should not be considered direct evidence of target binding or the mechanism of action. While the docking results provide useful structural insights into the predicted interaction between SAC and NF-κB, the biological effects of SAC are primarily supported by our *in vitro* signaling assays and *in vivo* asthma model, in which SAC consistently attenuated airway inflammation, modulated immune responses, and reduced PI3K/Akt/NF-κB signaling.

## 5 Conclusion

In conclusion, the present study demonstrated distinct differences in the protective effects of the three garlic-derived allyl sulfides against Der p-induced allergic asthma. SAC exhibited the strongest anti-inflammatory and immunomodulatory effects, DADS showed partial moderate activity, while DATS had no significant effect. SAC decreased airway inflammatory cell infiltration, restored the Th1/Th2 immune balance, modulated macrophage-associated cytokines, reduced allergen-specific IgE and IgG1 production, and was associated with reduced PI3K/Akt/NF-κB signaling. These findings indicate that SAC exerts strong protective effects against Der p-induced allergic asthma by regulating immune responses, suppressing airway inflammation, and preserving epithelial integrity. Both the *in vivo* and *in vitro* findings highlight SAC as the most promising natural candidate for preventing and managing allergic asthma and related airway inflammatory diseases.

## Data Availability

The raw data supporting the conclusions of this article will be made available by the authors, without undue reservation.
